# Heat’s Role in Solution Electrospinning: A Novel Approach to Nanofiber Structure Optimization

**DOI:** 10.1021/acs.langmuir.3c03919

**Published:** 2024-04-03

**Authors:** Michael Wildy, Wanying Wei, Kai Xu, John Schossig, Xiao Hu, Dong Choon Hyun, Wenshuai Chen, Cheng Zhang, Ping Lu

**Affiliations:** † Department of Chemistry and Biochemistry, Rowan University, Glassboro, New Jersey 08028, United States; ‡ Department of Physics and Astronomy, Rowan University, Glassboro, New Jersey 08028, United States; § Department of Polymer Science and Engineering, Kyungpook National University, Daegu 41566, South Korea; ∥ Key Laboratory of Bio-based Material Science and Technology, Ministry of Education, Northeast Forestry University, Harbin 150040, China; ⊥ Chemistry Department, Long Island University (Post), Brookville, New York 11548, United States

## Abstract

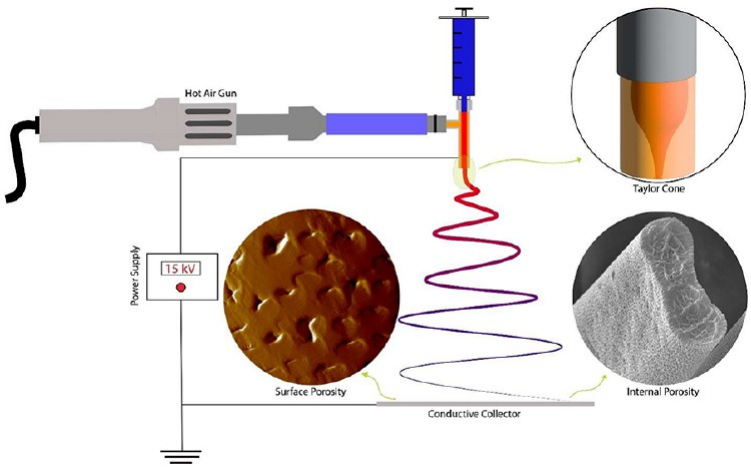

In this study, we explored an innovative application of heat-assisted solution electrospinning, a technique that significantly advances the control of phase separation in polystyrene (PS) fibers. Our experimental approach involved the use of direct heating and a convection air sheath applied through a coaxial needle, focusing on solvents with varying vapor pressures. This method enabled a detailed investigation into how solvent evaporation rates affect the morphology of the electrospun fibers. SEM and AFM measurements revealed that the application of direct heating and a heated air sheath offered precise control over the fiber morphology, significantly influencing both the surface and internal structure of the fibers. Additionally, we observed notable changes in fiber diameter, indicating that heat-assisted electrospinning can be effectively utilized to tailor fiber dimensions according to specific application requirements. Moreover, our research demonstrated the critical role of solvent properties, particularly vapor pressure, in determining the final characteristics of the electrospun fibers. By comparing fibers produced with different solvents, we gained insights into the complex interplay between solvent dynamics and heat application in fiber formation. The implications of these findings are far-reaching, offering new possibilities for the fabrication of nanofibers with customized properties. Furthermore, this could have profound impacts on various applications, from biomedical to environmental, where specific fiber characteristics are crucial. This study not only contributes to the understanding of phase separation in electrospinning but also opens avenues for further research on the optimization of fiber properties for diverse industrial and scientific applications.

## Introduction

Porous materials, encompassing a diverse range such as metal–organic frameworks (MOFs), activated carbon, biochar, porous silica, zeolites, and fibers, have garnered widespread attention due to their exceptional utility in sectors including energy storage, filtration, sensing, biomedical engineering, and catalysis.^1−8^ These materials are characterized by their high porosity and surface area, making them ideal for these applications. Nonwoven membranes, such as randomly orientated fiber mats, exemplify a unique subset of these materials.^9^ They possess intrinsic interfiber porosity resulting from the layering of numerous fiber layers, offering remarkable properties and performance that are increasingly recognized in various fields.^10^ The use of nanofibers, known for their high surface-to-volume ratio due to ultrafine diameters, further accentuates this aspect.^11^ Furthermore, these nonwoven membranes can also exhibit intrafiber (or internal) porosity, manifesting as pores on the surface or within the individual fibers.^12^ The presence of extremely small intrafiber pores can significantly amplify the surface area, thus enhancing the efficiency and capacity of these materials for diverse applications, ranging from advanced filtration systems to high-performance catalytic reactors.^13−16^ Electrospinning stands as a versatile electrohydrodynamic atomization technique for fabricating porous fiber mats, ranging from nano- to microscale fibers, utilizing a high voltage to transform various polymers into fibers.^17^ Predominantly, solution electrospinning is applied, which involves dissolving polymers in suitable solvents to create fiber-forming solutions.^18^ In contrast, melt electrospinning, a solvent-free approach, uses heat to liquefy polymers, enabling fiber stretching during electrospinning.^19^ Despite its potential, melt electrospinning’s complexity, the requirement for a high processing temperature to reach the melting point of polymers, and the tendency to produce larger fiber sizes (usually tens to several hundred micrometers) have limited its popularity compared to solution electrospinning.^20^ Furthermore, only a small portion of polymers melt without thermal degradation.^21^

Within solution electrospinning, many methods have been employed to control the fiber porosity. Phase separation, a critical phenomenon in this context, can occur due to the demixing of polymer and solvent in the liquid jet, especially during solvent evaporation.^22^ The employment of volatile solvents has proven effective in creating highly porous fibers.^23,24^ Another prevalent technique is the nonsolvent-induced phase separation (NIPS), where introducing a nonsolvent incapable of dissolving the polymer induces phase separation, resulting in polymer-rich and polymer-lean domains.^25−27^ This process, coupled with the rapid solvent evaporation under high-charge conditions in electrospinning, leads to the formation of intrafiber pores.^28^ Moreover, ambient relative humidity has been identified as a critical factor influencing the quality and morphology of electrospun fibers. This is particularly relevant because water vapor acts as a nonsolvent for hydrophobic polymers, introducing a unique dynamic in the fiber formation process.^29,30^ For hydrophobic polymers, the interaction between water vapor and the polymer solution is pivotal. When water vapor penetrates the liquid jet during electrospinning, it interacts with the polymer molecules, forming polymer lean domains.^31−33^ In our recent studies, the formation of porous structures in polyacrylonitrile (PAN) and polystyrene (PS) nanofibers was investigated using NIPS.^25^ Different from the traditional strategy to control the nonsolvent content in polymer solution or environment vapor, we utilized coaxial electrospinning to directly introduce nonsolvents such as water and ethylene glycol (EG) into polymer jets, effectively controlling phase separation and fabricating nanofibers *in situ*. We found that the intermolecular interactions between nonsolvents and polymers dictated the development of phase separation and porous structure. We also observed that the size and polarity of the nonsolvent molecules influenced this process. Furthermore, the choice of solvent, particularly in terms of evaporation kinetics, plays a pivotal role in phase separation, as evidenced by the differing porous structures formed when using rapidly evaporating solvents such as tetrahydrofuran (THF) compared to slower-evaporating solvents such as dimethylformamide (DMF).

Thermally induced phase separation (TIPS) represents another effective approach in the production of porous nanomaterials, finding particular utility in electrospinning to generate porous nanofibers.^34,35^ The underlying mechanism of TIPS hinges on the temperature differential between the nanofibers and their surrounding environment.^36^ Traditional methods to enhance this differential involve either heating or cooling strategies. In the realm of electrospinning, active solution heating emerges as a superior method.^37,38^ This approach not only creates a larger temperature difference but also beneficially impacts the polymer solution by enhancing solubility and reducing viscosity.^39^ Typically, solution heating in electrospinning is achieved using a syringe jacket heater.^40^ However, this method faces challenges such as rapid and significant temperature drops at the needle portion, attributable to the low feed rate and the small volume of solution with a large surface area exposed to the environment.^41^ To address this, alternative techniques like infrared emitters and lasers were utilized to heat the needle and liquid jet.^42,43^ Despite their effectiveness, these methods require precise focusing on the needle tip, making them susceptible to inefficiencies due to slight misalignments.^44,45^ In contrast to melt electrospinning, which requires heating to the melting point of polymers, the heating in solution electrospinning does not necessitate such high temperatures. In this work, we developed an innovative heated air sheath using a coaxial needle in addition to direct convection heating. As a result, the heating was uniformly distributed throughout the needle portion, concentrating specifically on the small volume of polymer solution within the needle for fast and efficient heating. Furthermore, this targeted approach effectively prevented the extensive volume expansion that typically occurs when heating is applied to the entire syringe. Our novel approach facilitated the control of polymer phase separation and internal porosity during the electrospinning process. By heating the polymer jet, we effectively modulated the solvent evaporation rate and the extent of water vapor penetration, demonstrating efficacy with polymers in both low-volatility (i.e., DMF) and high-volatility (i.e., THF) solvents. Our coaxially heated air sheath and direct heating techniques, applied to the Taylor cone in the electrospinning setup, allowed for unparalleled control over phase separation. This heat-assisted electrospinning technique, as applied to PS in both DMF and THF, resulted in a controllable surface morphology, internal structure, and fiber diameter, marking a significant advancement in the field of nanofiber fabrication.

## Experimental Section

### Chemicals and Materials

High molecular weight polystyrene (PS) with a molecular weight (*M*_w_) of 350000 and a number-average molecular weight (*M*_n_) of 170000 was sourced from Sigma-Aldrich. This particular grade of PS was chosen for its suitability in electrospinning processes due to its favorable flow and viscoelastic properties. The polymer was used as the primary constituent for preparing the electrospinning solutions. For the solvent system, we utilized anhydrous *N*,*N*-dimethylformamide (DMF) and tetrahydrofuran (THF), both with a purity of ≥99.9%. These chemicals were obtained from VWR International. DMF was selected for its ability to effectively dissolve high molecular weight polystyrene, ensuring a homogeneous solution ideal for electrospinning. Similarly, THF, known for its rapid evaporation rate, was used to explore the effects of the solvent dynamics on the electrospinning process. Both solvents were used as received without any additional purification.

### Heat-Assisted Electrospinning

**Figure 1:**
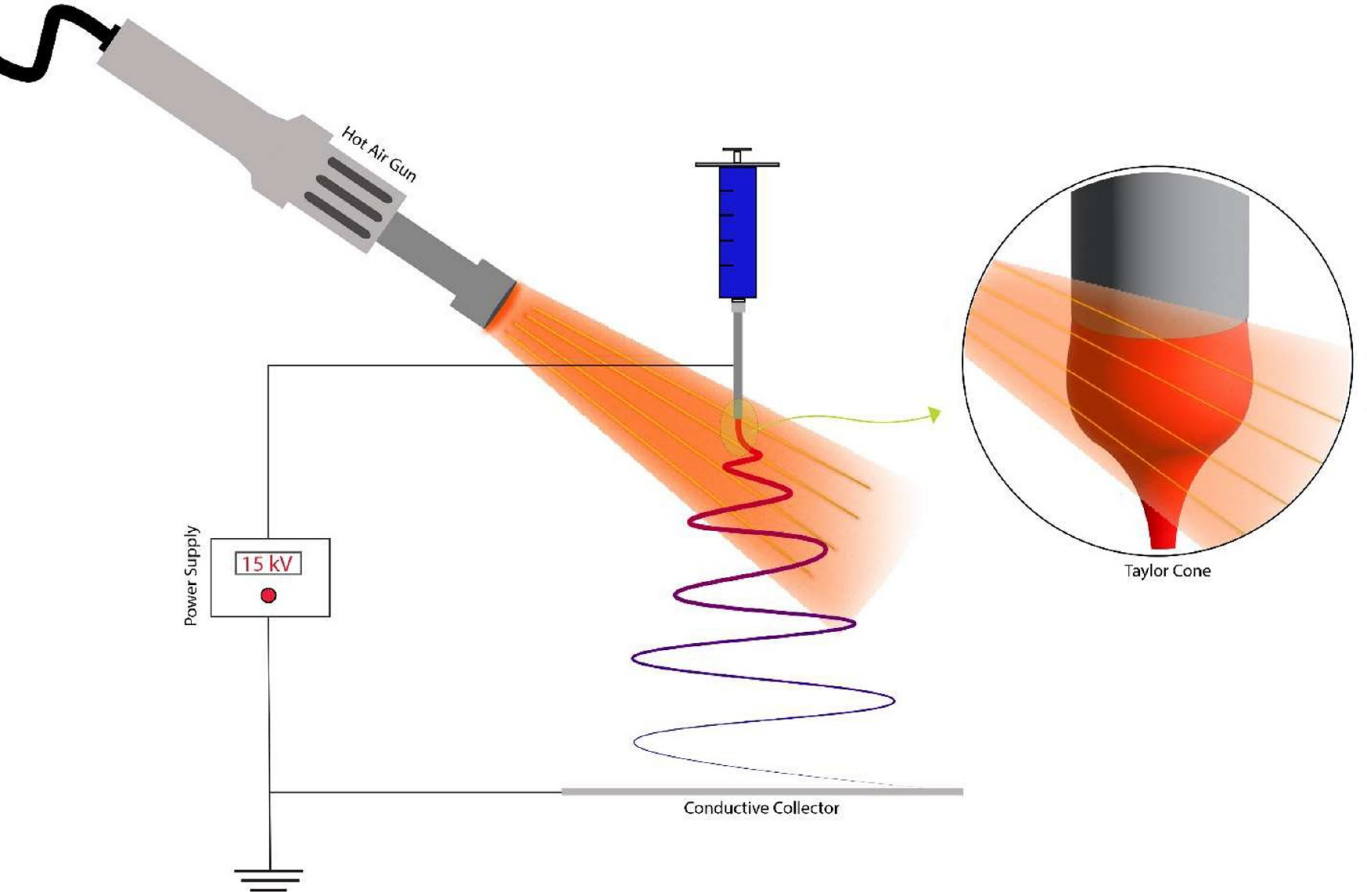
Direct convection heating-assisted electrospinning setup.

**Figure 2:**
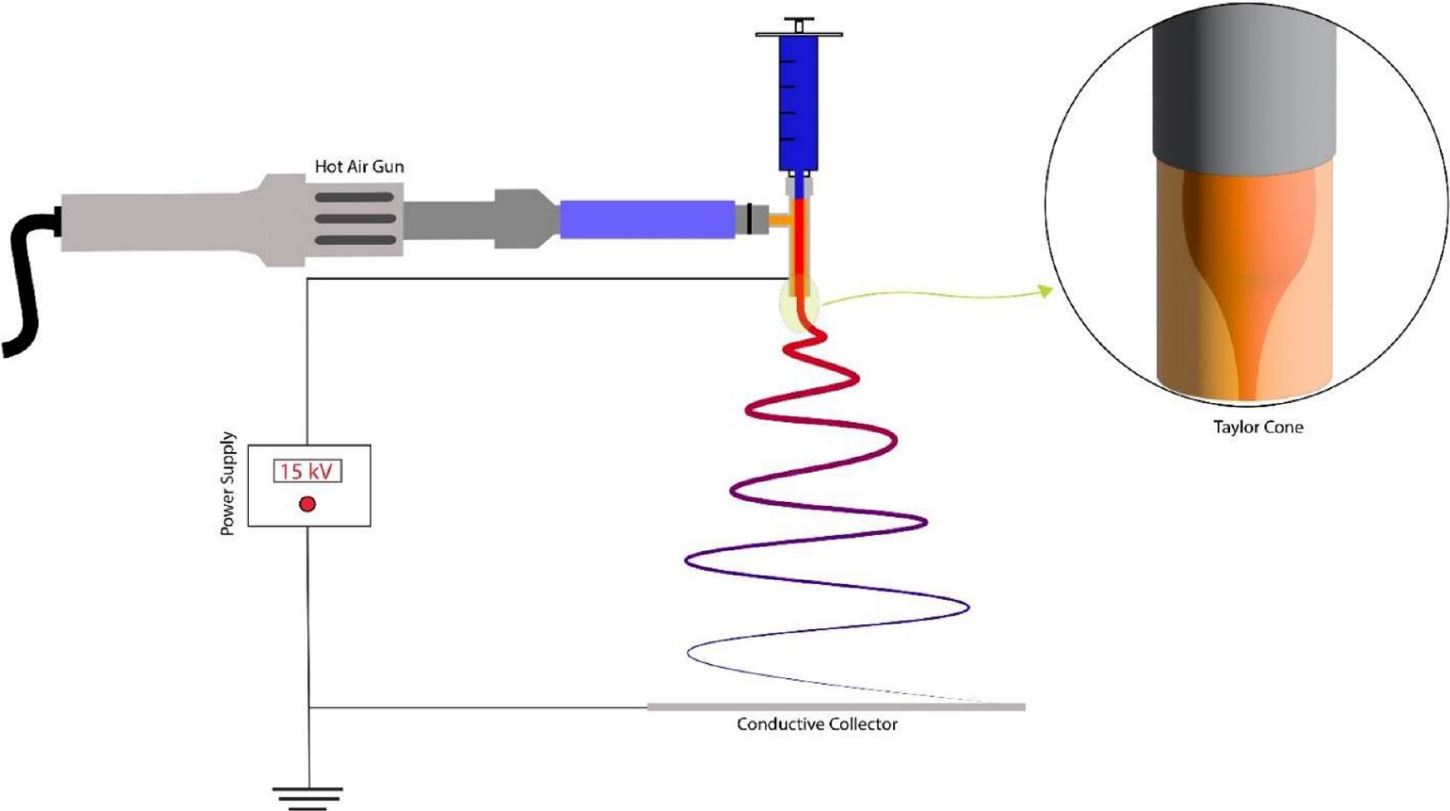
Heated air sheath-assisted electrospinning setup.

For our experiments, polymer solutions comprising 20 wt % PS in anhydrous DMF or THF were meticulously prepared. These solutions were stirred continuously for 24 h to ensure thorough mixing and homogeneity. The PS solution in DMF was electrospun by using a 22-gauge blunt needle. We employed a programmable syringe pump (Legato 110, KD Scientific) for precise control of the solution flow through the electrospinning spinnerets. Figure 1 presents a detailed schematic illustration of the direct convection heating-assisted electrospinning setup. In this setup, a stream of heated air, supplied by an ATTEN ST-862D hot air gun, was positioned 6 cm away and 45° from the needle tip. The position and angle of the hot air gun were optimized to achieve the best results. We experimented with various hot air gun temperatures—220, 260, 300, and 340 °C—to fabricate distinct samples labeled PS-DMF-220, PS-DMF-260, PS-DMF-300, and PS-DMF-340, respectively. In the coaxial electrospinning process involving THF as the solvent, Figure 2 illustrates the heated air sheath-assisted electrospinning setup. Here, the polymer solution was fed into the inner needle of a 22-gauge inner/18-gauge outer concentric needle assembly. The outer needle was equipped with a hot air gun, connected via a short segment of high-temperature-resistant silicone tubing encased in a rubber insulation sheath. The hot air gun temperatures set at 150, 200, and 250 °C were used to produce samples PS-THF-150, PS-THF-200, and PS-THF-250, respectively. As a control, reference PS fibers were also fabricated without the application of a hot air stream (PS-DMF, PS-THF). In a typical electrospinning experiment, a high-voltage DC power supply (ES30P-5W, Gamma High Voltage Research) was utilized to generate a 15 kV potential, which created the Taylor cone and facilitated the ejection of the liquid polymer jet. This process stretched the PS jet toward a grounded collector, forming the fiber membranes. The needle tip-to-collector distances were set at 25 cm for PS in DMF and 15 cm for PS in THF. To maintain consistent electrospinning conditions, we regulated the laboratory environment using central air conditioning and an industrial-sized humidifier/dehumidifier. This setup ensured a stable temperature of 20 ± 2 °C and a relative humidity of 50 ± 3%. Post-electrospinning, the samples were subjected to vacuum drying at ambient temperature for a minimum of 24 h prior to further analysis.

### Characterization

The surface morphology and interior structure of the nanofibers were meticulously analyzed by using high-resolution field-emission scanning electron microscopy (SEM, Apreo model by FEI). To prepare for cross-sectional imaging, the nanofibers were initially frozen in liquid nitrogen. This rapid freezing technique is critical as it preserves the fiber structure intact for accurate assessment. Following this, the frozen fibers were carefully stretched to fracture, thus revealing their internal architecture. The fractured samples were then dried in a forced-air oven at 60 °C to remove any residual moisture, ensuring clear and distortion-free imaging. Prior to SEM examination, the nanofiber samples underwent a gold sputter-coating process for 120 s. This step is essential for enhancing the electrical conductivity of the fibers, thereby preventing charging effects during SEM imaging and ensuring high-quality, artifact-free images. In the SEM analysis, we meticulously adjusted the settings to optimize the image clarity and detail. These settings included a working distance of 6 mm, an accelerating voltage of 10 kV, and a beam current of 0.40 nA. These parameters were carefully chosen to provide a balance between the resolution and sample integrity. For quantitative analysis, ImageJ software (version 1.54d, NIH) was employed to calculate the average diameters of the nanofibers. This analysis was based on at least 50 measurements of representative fibers across various SEM images, ensuring statistical significance and reliability. Additionally, OriginPro software (OriginLab) was utilized for the statistical analysis and graphical representation of the measurement data. The topographical analysis of the nanofiber samples was performed using an advanced Bruker Dimension XR scanning probe microscope system (Santa Barbara, CA). Prior to AFM imaging, the samples were meticulously prepared to ensure an optimal imaging quality. A dilute suspension of the nanofibers in ethanol (approximately 0.01% concentration) was prepared. Several drops of this suspension were then gently deposited onto a freshly cleaved mica surface. These surfaces, comprising the highest grade V1 mica discs of 12 mm diameter, were sourced from Electron Microscopy Sciences. The mica’s atomically flat surface is ideal for AFM analysis, ensuring an accurate representation of the sample’s topography. The nanofiber-laden mica discs were then left undisturbed to dry completely, forming a thin, evenly distributed layer of fibers suitable for high-resolution scanning. AFM imaging was conducted under ambient conditions, with the room temperature and humidity carefully monitored to maintain consistency. The tapping mode was employed for these scans, a technique chosen for its ability to provide high-resolution images while minimizing tip–sample interaction forces that could potentially distort the nanofibers. We utilized an OTESPA-R3 standard silicon probe from the Olympus Corp. for the scanning. These probes, featuring a tip radius of less than 10 nm, a spring constant of 26 N/m, and a resonance frequency of 300 kHz, are specifically designed for high-resolution imaging. During the imaging process, a scanning rate of 1 Hz was maintained, and the resolution was set at 512 pixels × 512 pixels, ensuring a detailed and accurate representation of the nanofiber topography. The resultant images were then processed by using NanoScope Analysis 3.00 software. This software facilitated a detailed section analysis, providing a comprehensive understanding of the nanofiber surface structure.

## Results and Discussion

**Figure 3:**
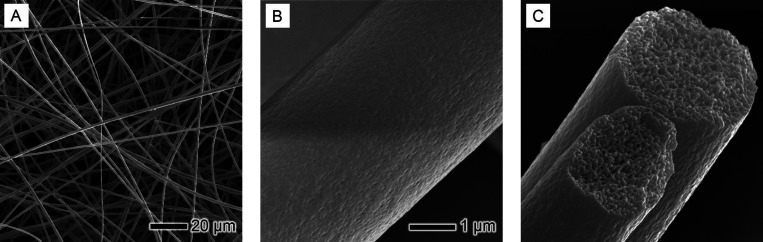
Detailed SEM analysis of PS fibers electrospun in DMF: (A) an overview of the fiber mat, showcasing the general morphology and distribution of fibers; (B) zoom-in of the surface texture of individual fibers, highlighting the uniformity and any microscale features; and (C) a cross-sectional view of the fibers, revealing the internal structure and porosity. The scale bar in (B) is applicable to both (B) and (C).

The electrospinning of PS in DMF yielded uniform fibers characterized by minimal defects such as beading. The application of a directed hot air stream during electrospinning of PS in DMF facilitated the control of phase separation. Our prior studies have indicated that the diffusion of environmental water vapor contributes to the formation of a highly porous interior within PS nanofibers when using DMF as the solvent.^31^ The inherent nonpolarity of polystyrene juxtaposed with the high polarity of water results in minimal molecular interactions. This disparity in polarity aided in the formation of distinct domains, varying in polymer concentration due to the incompatibility of PS and water molecules. The miscibility of DMF and water further encouraged the demixing of polymer-lean and polymer-rich domains. Water vapor penetration into the electrospun PS fibers is facilitated when employing a low-volatility solvent like DMF.^25^ At high humidity levels, water vapor can saturate the atmospheric side of the jet–air interface, leading to the formation of a solidified PS sheath layer and the subsequent demixing of PS, DMF, and water mixture in the core region. SEM images of PS-DMF fibers (Figure 3) revealed uniformity in the fiber diameter and distinctive pores. Particularly, a substantial degree of internal porosity was evident, indicative of the phase separation process stemming from the demixing of PS and DMF due to water vapor diffusion.

**Figure 4:**
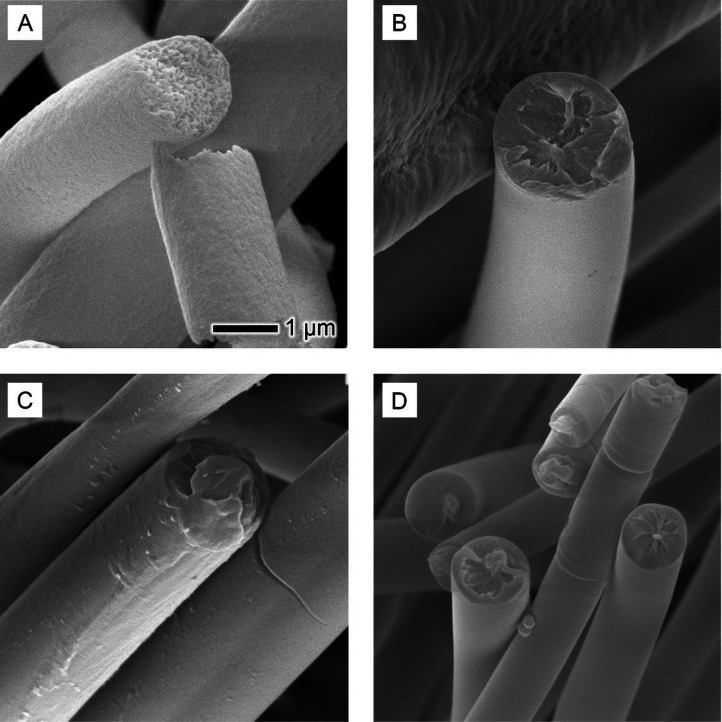
Cross-sectional SEM images showcasing the effect of varied heating temperatures on PS-DMF fibers: (A) internal structure of fibers electrospun at 220 °C (PS-DMF-220), illustrating the degree of porosity and phase separation at this temperature; (B) fibers electrospun at 260 °C (PS-DMF-260), showing changes in internal morphology compared to lower temperatures; (C) fibers electrospun at 300 °C (PS-DMF-300), highlighting further alterations in the fiber structure indicative of temperature impact; and (D) fibers electrospun at 340 °C (PS-DMF-340), demonstrating the effects of the highest temperature on fiber porosity and internal configuration. The scale bar in (A) applies to all images.

The introduction of heated air during electrospinning effectively minimized the phase separation caused by water vapor penetration. In samples electrospun with direct heating at temperatures ranging from 220 to 340 °C (Figure 4), a trend of decreasing phase separation with increasing temperature was observed. Cross-sectional images enabled a detailed examination of interior phase separation. At 220 °C, porous PS fibers similar to those in nonheated samples were noted. Elevating the temperature to 260 °C led to a mix of solid and porous fibers, suggesting the ability of heated air to modulate phase separation. A notable thickening of the outer sheath layer, as seen in the SEM images (Figure 4B), indicates that heating altered the solvent evaporation rate at the polymer–atmosphere interface. With further temperature increases to 300 and 340 °C, a significant reduction in formation of internal pores was observed, with the latter temperature resulting in fibers of smaller diameter and predominantly solid interiors. These findings underscore the capacity of heated air to precisely control the degree of phase separation and porous interior in PS fibers. High humidity conditions can lead to a prolonged solvent evaporation process in electrospun fibers, particularly when the electrospinning solvent is miscible with water.^46^ The heat-accelerated solvent evaporation rate likely facilitated earlier formation of the PS sheath layer, reducing water vapor penetration. Additionally, the heated environment around the Taylor cone likely expedites solvent evaporation, at both the surface and interior of the fibers, curtailing the period for water and solvent mixing/demixing and minimizing water availability for phase separation.^47^ Furthermore, the nanofibers were heated to a temperature above the glass transition but below the polymer melting point temperature, allowing polystyrene molecules to adopt a more relaxed configuration. Therefore, the ability of polymer-lean domains to form was greatly reduced and fibers with nonporous internal structure were formed when temperature was increased.^48^

**Figure 5:**
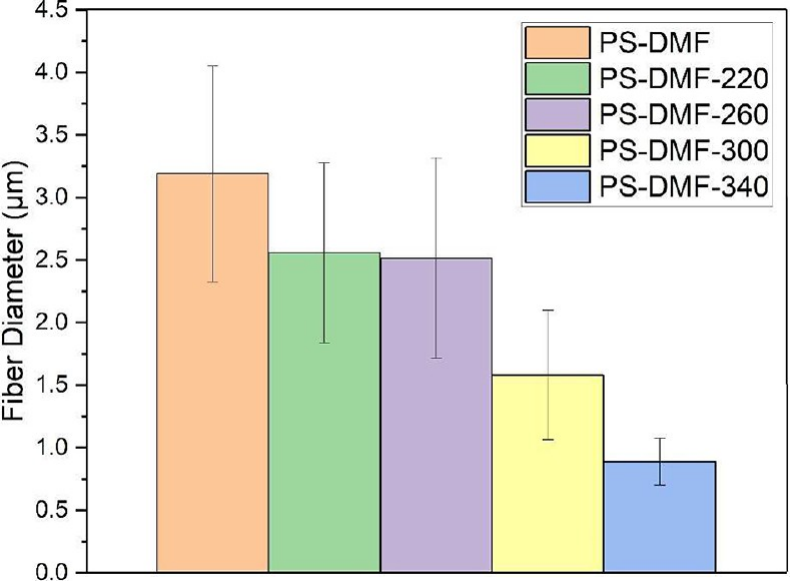
Comparative analysis of fiber diameters in direct convection heating-assisted electrospun PS-DMF fibers. This figure presents a graphical representation of the variation in fiber diameters across different temperatures. Each data point or bar represents the average diameter measured under specific heating conditions.

The data presented in Figure 5 clearly demonstrate a trend of decreasing average fiber diameter with increasing temperature of the heated air stream during the electrospinning process. For the PS-DMF fibers electrospun without the application of heat, the average diameter was recorded at 3.189 ± 0.862 μm. In contrast, the diameters of the direct convection heating-assisted electrospun PS-DMF fibers showed a distinct reduction across the temperature spectrum. Specifically, the fiber diameters measured 2.558 ± 0.721 μm for PS-DMF-220, 2.514 ± 0.800 μm for PS-DMF-260, 1.579 ± 0.518 μm for PS-DMF-300, and, most notably, 0.888 ± 0.188 μm for PS-DMF-340. This decreasing trend in fiber diameter with higher processing temperatures can be attributed to several factors. Primarily, the increase in temperature facilitated a more elongated stretching of the polymer jet due to decreased viscosity and surface tension, contributing to finer fiber formation. Additionally, the higher temperatures enhanced the evaporation rate of the solvent, leading to a more rapid solidification and drawing of the polymer jet and consequently, thinner fibers.^49^ Furthermore, the uniformity of the fibers also showed significant improvement at higher temperatures, particularly at 340 °C, as indicated by the marked decrease in the standard deviation of the fiber diameter measurements. This improvement in uniformity suggests that the controlled application of heat not only refines the fiber diameter but also contributes to a more consistent fiber production process. This aspect is particularly important for applications where uniform fiber dimensions are critical, such as in filtration or tissue engineering scaffolds, where the consistency in the pore size and fiber morphology directly impacts the functional performance of the material. These findings highlight the effectiveness of heat-assisted electrospinning in precisely controlling fiber dimensions, emphasizing its potential for tailoring nanofiber properties to specific application requirements.

**Figure 6:**
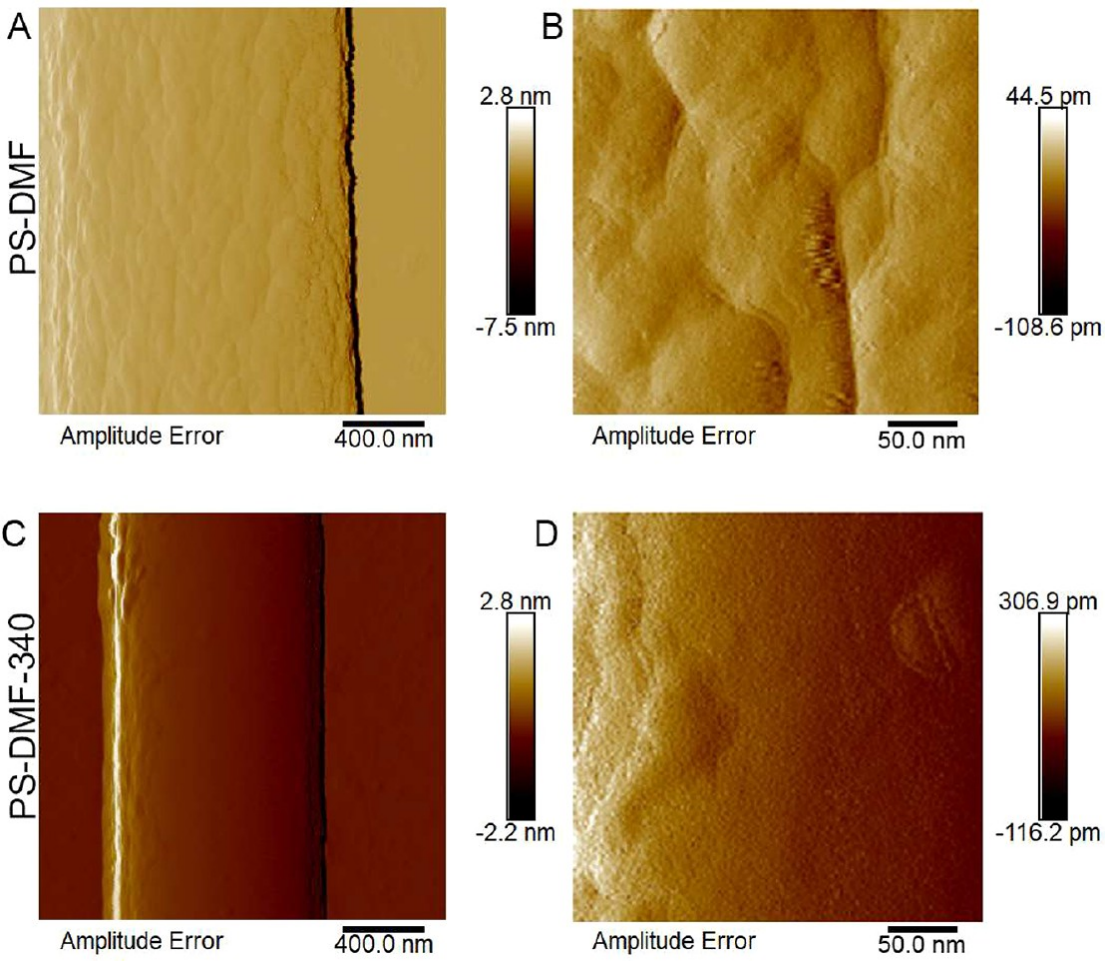
AFM images illustrating the surface topography of PS-DMF fibers: (A, B) surface texture of PS-DMF fibers electrospun without heat assistance, highlighting the inherent morphology and surface features under standard electrospinning conditions; (C, D) surface characteristics of PS-DMF fibers electrospun with heat assistance at 340 °C (PS-DMF-340), showcasing the impact of high-temperature treatment on fiber surface structure.

The AFM images in Figure 6 provide compelling insight into how the surface morphology of PS fibers was altered by heat-assisted electrospinning. Generally, PS fibers electrospun at a relative humidity (R.H.) of about 50% exhibit a formation of small pores on their surface, likely due to the phase separation processes influenced by environmental conditions (e.g., water vapor condensation).^31^ The AFM images of PS-DMF and PS-DMF-340 nanofibers reveal significant differences in surface texture attributable to the temperature variations during the electrospinning process. In the case of PS-DMF fibers electrospun without heat assistance, the images (Figure 6A,B) show a relatively rougher surface with a more pronounced porosity. This is indicative of the phase separation occurring at the fiber surface, likely facilitated by the interaction between the polymer and environmental water vapor during the electrospinning process. Conversely, the PS-DMF-340 fibers, which were electrospun with the application of heat at 340 °C, display a markedly different surface morphology (Figure 6C,D). These fibers exhibited a smoother surface, with a significantly reduced number of pores observed. The minimization of the surface phase separation during the heat-assisted electrospinning process was evident. The elevated temperature likely accelerated solvent evaporation, reducing the time available for phase separation to occur at the fiber surface. As a result, the fibers cooled and solidified more rapidly, leading to a decrease in the apparent surface porosity. This finding could have important implications for applications where surface smoothness and reduced porosity are desirable, such as in certain biomedical applications where smoother fiber surfaces may be beneficial for cell attachment and growth. The ability to control surface morphology through temperature modulation in the electrospinning process offers a versatile tool for tailoring fiber properties to specific application needs.

**Figure 7:**
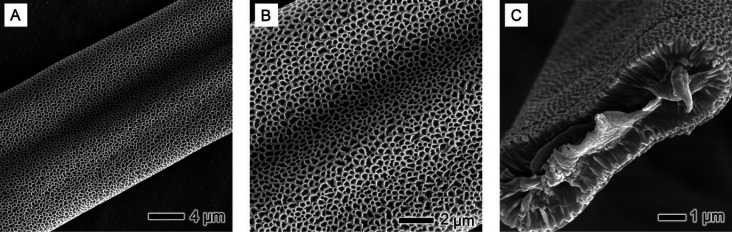
SEM images highlighting the surface and cross-sectional morphology of PS-THF fibers. (A, B) A detailed view of the surface texture of the fibers, revealing the microscale features and topography characteristic of fibers electrospun using THF as the solvent. These images provide insights into the surface intricacies and fiber uniformity. (C) A cross-sectional view of the fibers, illustrating their internal structure and providing a comprehensive understanding of the fiber morphology from a different perspective.

The utilization of THF as the solvent in the electrospinning process significantly influenced the stability of the Taylor cone and the continuity of fiber flow, resulting in frequent clogging and interruption of fiber production. This instability can be attributed to the high vapor pressure of THF (19.07 kPa at 20 °C), which leads to a rapid rate of solvent evaporation, significantly surpassing that of DMF. This rapid evaporation poses challenges in maintaining a consistent electrospinning process, as the solvent’s quick departure from the liquid jet can disrupt the formation of a stable Taylor cone and the subsequent liquid jet. Compared to the electrospinning of PS in DMF, the fibers produced with THF exhibited markedly different morphologies, as revealed in Figure 7. The PS-THF fibers were characterized by a larger size and a distinctive flat, ribbon-like shape. This morphology is likely a consequence of the rapid evaporation rate of THF, coupled with the high surface tension of the polymer solution. The large fiber size and solvent volatility also contributed to condensation of water vapor on the fiber surface during the electrospinning process. Once collected, this condensed water vapor evaporated, leaving behind large surface pores, indicative of a phase separation process influenced by environmental humidity.^25,31,50^

**Figure 8:**
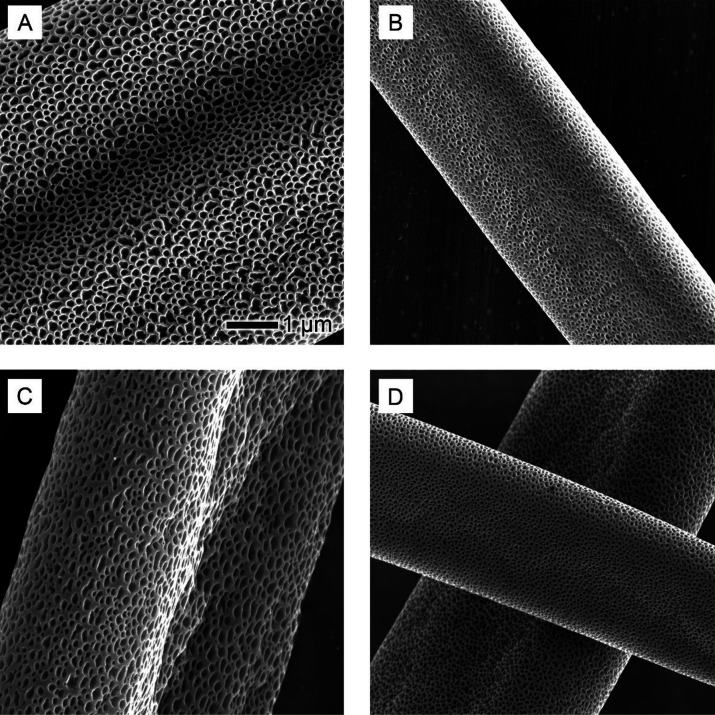
SEM images showcasing the surface morphology of PS-THF fibers under various heat-assisted electrospinning conditions: (A) surface texture of PS-THF fibers electrospun without heat assistance, illustrating the baseline morphology; (B–D) surface characteristics of PS-THF fibers electrospun with heat assistance at 150 °C (PS-THF-150), 200 °C (PS-THF-200), and 250 °C (PS-THF-250), respectively. The scale bar in (A) applies to all images.

In Figure 8, the surface morphology of PS-THF fibers electrospun under various heating conditions is illustrated. The application of a heated air sheath notably impacted the phase separation during the electrospinning of PS in THF. Heat-assisted PS-THF fibers exhibited a surface morphology characterized by shallow pores, similar to nonheated samples. However, the absence of pronounced phase separation suggests that the penetration of condensed water vapor was minimal under the heated conditions. Interestingly, heating was found to increase the apparent internal porosity, a phenomenon that can be attributed to the enhanced mobility of polymer chains at elevated temperatures, facilitating the formation of more porous structures.

**Figure 9:**
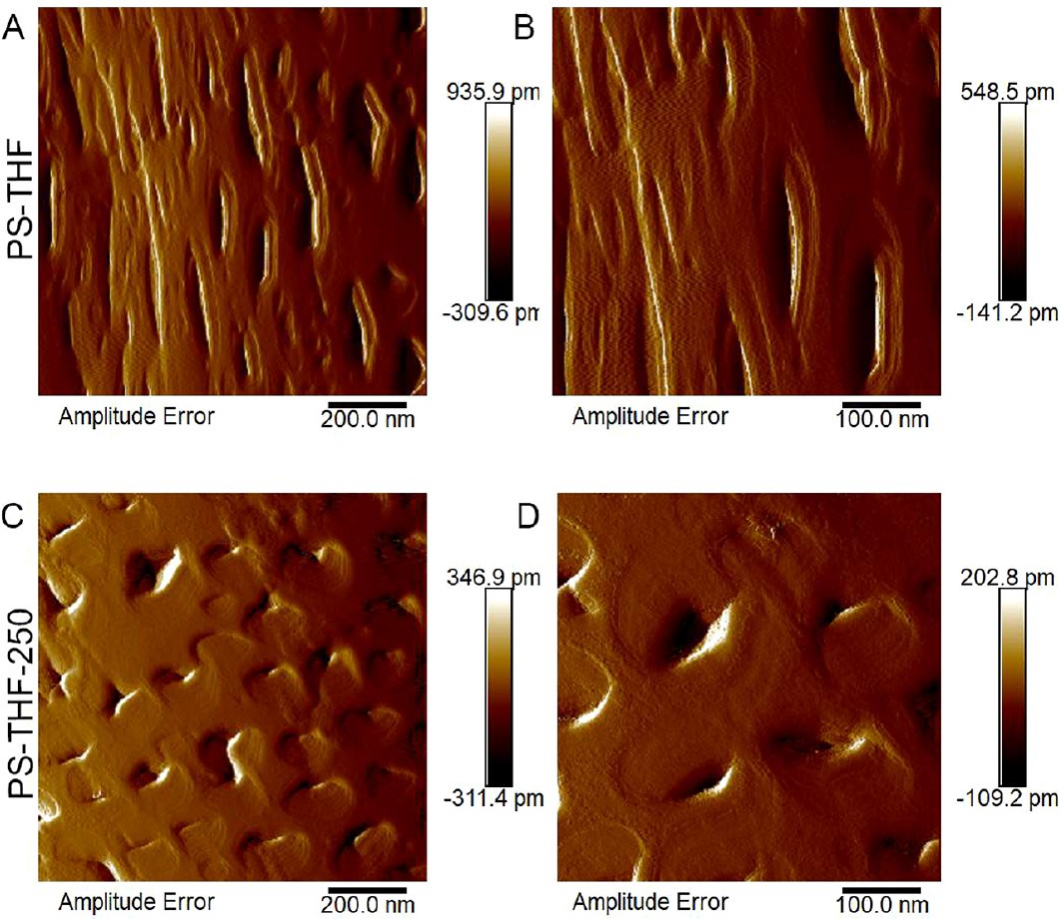
AFM images comparing the surface topography of PS-THF fibers with and without heat assistance: (A, B) surface morphology of PS-THF fibers electrospun under standard conditions, showcasing their inherent textural characteristics; (C, D) surfaces of PS-THF fibers electrospun with heat assistance at 250 °C (PS-THF-250), highlighting the changes in surface topography induced by the application of heat.

The AFM images in Figure 9 shed light on the nuanced effects of using a heated air sheath during the electrospinning of PS-THF fibers on their surface morphology. The PS-THF fibers, electrospun without heat assistance, exhibited elliptical-shaped pores on their surfaces. This elliptical pore shape is indicative of the dynamic process of water vapor condensation and subsequent evaporation during the fiber stretching and drawing phases of electrospinning. The elongated pore shape suggests a slower evaporation rate, where water vapor has enough time to condense and then slowly evaporate, leaving behind distinct pore structures. In stark contrast, the PS-THF-250 fibers, electrospun with the application of heat at 250 °C, presented significantly different pore morphology. The pores on these fibers were not elongated, implying that the application of heat through the air sheath effectively accelerated solvent evaporation, thus minimizing the condensation of water vapor. This rapid evaporation likely “freezes” the pore structures in place more quickly, resulting in smaller, less elongated pores. The heat from the air sheath also presumably reduced the relative humidity at the fiber–air interface, altering the conditions under which solvent evaporation and pore formation occur. These observations underscore the critical role of temperature control in manipulating the microstructural characteristics of the electrospun fibers. By adjusting the heat applied during electrospinning, one can significantly influence the evaporation kinetics of the solvent and, consequently, the morphology of the fiber surface.

**Figure 10:**
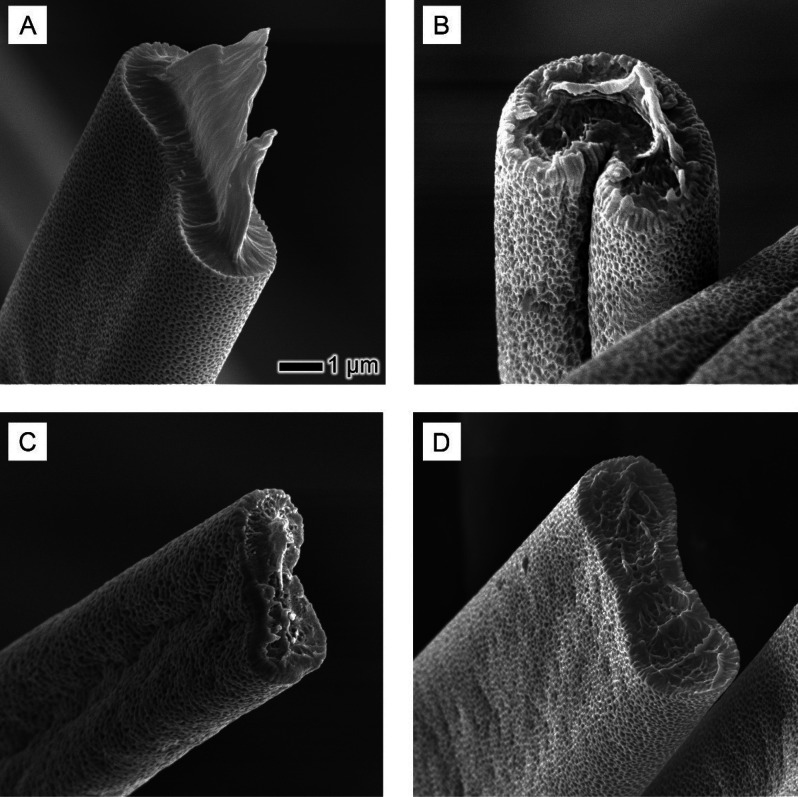
Cross-sectional SEM images of PS-THF fibers under various heat-assisted electrospinning conditions: (A) cross-sectional view of PS-THF fibers electrospun without heat assistance, showing the baseline internal structure; (B–D) cross sections of PS-THF fibers electrospun with heat assistance at 150 °C (PS-THF-150), 200 °C (PS-THF-200), and 250 °C (PS-THF-250), respectively. The scale bar in (A) applies to all images.

The cross-sectional SEM images in Figure 10 provide pivotal insights into the internal structure of PS-THF fibers, particularly highlighting the effect of the temperature on their apparent internal porosity. A clear trend was observed: as the temperature of the heated air sheath increased, the frequency of internal pores of the fibers correspondingly escalated. This trend is a significant finding, as it reveals the intricate interplay between temperature and the internal microstructure of electrospun fibers. In the absence of heat (PS-THF fibers), the fibers predominantly exhibit a solid internal polymer network (Figure 10A). This dense structure can be attributed to the relatively slow evaporation of the solvent under ambient conditions, allowing the polymer chains to align and solidify into a compact arrangement. However, the introduction of a heated air sheath dramatically altered this morphology. With the application of heat, there was an evident increase in the polymer–solvent demixing, likely driven by the thermally induced phase separation (TIPS) mechanism. This phenomenon occurred as the elevated temperature boosted the solvent evaporation rate, creating an environment conducive for the polymer chains to segregate and form distinct porous structures. The increase of pores within the fibers at higher temperatures (150, 200, and 250 °C), as clearly shown in Figure 10B–D, suggests a more extensive demixing of polymer and solvent. This enhanced demixing is likely a result of the elevated temperatures accelerating solvent evaporation, leading to rapid phase separation before the polymer chains can fully align and solidify. The resultant internal structure was one with an increased number of void spaces, forming a porous network within the fiber.

This apparent porosity is not merely a physical alteration; it has profound implications for the functional properties of the fibers. Porous structures can significantly increase the surface area of the fibers, enhancing their potential utility in applications such as filtration, where larger surface areas can improve filtering efficiency, or in tissue engineering scaffolds, where porosity is crucial for cell infiltration and nutrient transport.^51−53^ Moreover, the ability to control the degree of porosity through temperature modulation provides a powerful tool for tailoring the properties of electrospun fibers to specific application needs.^52−56^ By adjusting the heat applied during the electrospinning process, it is possible to fine-tune the internal structure of the fibers, thereby customizing their mechanical and functional characteristics.^57^ This level of control opens up new avenues for the development of specialized nanofiber-based materials with tailored properties for various advanced applications.^54−57^

## Conclusions

In this study, we demonstrated a novel technique for the fabrication of nanofibers using heat-assisted solution electrospinning. Our approach, involving the use of direct convection heating and a heated air sheath applied through a coaxial needle, proved to be instrumental in controlling phase separation in PS fibers. This method enabled us to effectively manipulate the morphology of electrospun fibers with significant influence on their surface and internal structures. We revealed that the application of heated air significantly impacted the phase separation process, evident in the fibers electrospun from DMF and THF solvents. For PS-DMF fibers, the use of direct convection heating resulted in fibers with uniform diameters and a distinct porous structure, indicating precise control over the fiber morphology. Notably, increasing the temperature led to a reduction in the average fiber diameter, enhancing the uniformity of the fibers. This finding is particularly crucial for applications requiring consistent fiber dimensions, such as in filtration and tissue engineering scaffolds. In the case of PS-THF fibers, we observed a trend of an increased internal porosity with higher temperatures. This increase in apparent porosity can be attributed to the enhanced polymer–solvent demixing facilitated by the thermally induced phase separation mechanism. Our study also underscored the role of temperature in controlling the evaporation kinetics of solvents, thereby affecting the fiber surface morphology. The AFM and SEM analyses provided detailed insights into the microstructural changes and surface topographies under different electrospinning conditions. By precisely controlling the temperature during electrospinning, we were able to tailor the internal and surface properties of the fibers, offering vast potential for the fabrication of nanofibers with customized properties. Furthermore, this study contributes significantly to understanding phase separation in electrospinning, providing valuable insights into the dynamics of solvent evaporation and polymer morphology. It paves the way for future research aimed at optimizing fiber properties for diverse industrial and scientific applications.
